# Assessing the impacts of genetic defects on starch metabolism in *Arabidopsis* plants using the carbon homeostasis model

**DOI:** 10.1098/rsif.2023.0426

**Published:** 2023-11-29

**Authors:** Shuichi N. Kudo, Carolina C. M. Bello, Anthony Artins, Camila Caldana, Akiko Satake

**Affiliations:** ^1^ Graduate School of Systems Life Science, Kyushu University, Fukuoka 819-0395, Japan; ^2^ Department of Biology, Faculty of Science, Kyushu University, Fukuoka 819-0395, Japan; ^3^ Max Planck Institute of Molecular Plant Physiology, Golm/Postdam 14476, Germany

**Keywords:** mathematical model, circadian clock, photoperiod, starch metabolism, sucrose signalling, Bayesian inference

## Abstract

Starch serves as an important carbon storage mechanism for many plant species, facilitating their adaptation to the cyclic variations in the light environment, including day–night cycles as well as seasonal changes in photoperiod. By dynamically adjusting starch accumulation and degradation rates, plants maintain carbon homeostasis, enabling continuous growth under fluctuating environmental conditions. To understand dynamic nature of starch metabolism at the molecular level, it is necessary to integrate empirical knowledge from genetic defects in specific regulatory pathways into the dynamical system of starch metabolism. To achieve this, we evaluated the impact of genetic defects in the circadian clock, sugar sensing and starch degradation pathways using the carbon homeostasis model that encompasses the interplay between these pathways. Through the collection of starch metabolism data from 10 *Arabidopsis* mutants, we effectively fitted the experimental data to the model. The system-level assessment revealed that genetic defects in both circadian clock components and sugar sensing pathway hindered the appropriate adjustment of the starch degradation rate, particularly under long-day conditions. These findings not only confirmed the previous empirical findings but also provide the novel insights into the role of each gene within the gene regulatory network on the emergence of carbon homeostasis.

## 1. Introduction

The rotation and revolution of the Earth generate periodic change of light environments such as day–night cycles and seasonal changes in photoperiod. For photosynthetic organisms like plants, the adjustment of their physiological states to periodic fluctuations of light environments is crucial, as their survival, growth and reproduction rely on the photoassimilates synthesized solely during light period. To adapt to the periodic fluctuations of light environments, many plant species have evolved the ability to store a portion of the photoassimilates as transient starch in leaves during light period and to degrade it into sugars for energy provision during dark period in order to maintain carbon (C) homeostasis [1,2]. The rate of starch accumulation and degradation responds to changes in photoperiod in *Arabidopsis* [1,3–5]. When the photoperiod is short, plants increase the starch accumulation rate and decrease the starch degradation rate compared with longer photoperiods in order to prevent sugar depletion during the dark period. The dynamic adjustment of starch metabolism in response to changes in photoperiod can be advantageous for plants to grow continuously under fluctuating environmental conditions [6,7].

Previous experimental and theoretical studies have identified the importance of the circadian clock in the regulation of starch metabolism. Clock mutants of *Arabidopsis thaliana* with shorter or longer circadian period are unable to properly modulate the rate of starch degradation, resulting in C depletion or excess at the end of night [5]. For instance, the mutant *circadian clock associated*/*late elongated hypocotyl* (*cca1/lhy*) with a free running period of 17 h [8] exhausts its starch reserves earlier than wild-type, resulting in the induction of starvation marker genes [5]. This appears to be a consequence of an earlier onset in starch degradation (i.e. ZT9-12) [9]. By contrast, the long period mutant *pseudo-response regulator 7/9* (*prr7/9*) (a free-running period of 29.4 h [10]) delays the onset of starch degradation, resulting in continuous starch accumulation throughout the day [9]. More recently, the use of a triple mutant with impaired clock functions, *cca1/lhy/elf3*, which also lacks the *EARLY FLOWERING3* (*ELF3*), unravelled that plants with a dysfunctional clock are unable to adjust the starch accumulation rate, but they can still adjust the pace of starch degradation [11]. This means that the circadian oscillator might operate in a semi-autonomous mode to integrate temporal information in a complex network [11].

A crucial signal conveyed in this complex network is the sugar status. When C availability surpasses the growth capacity, sugar signals are incorporated into the regulation of pacing starch degradation. One example has emerged from the role of the sugar signal trehalose 6-phosphate (T6P) in regulating starch degradation in plants under conditions of excess C availability, such as in the sucrose efflux protein mutant *sweet11*/*12* [12] or genetic manipulation of T6P levels [13–15]. Modulation of Snf1-related kinase (SnRK1) activity also alters the sugar signalling by influencing how sucrose signals are conveyed into T6P, which in turn negatively impacts SnRK1 activity along the diel cycle, indirectly affecting starch turnover [16,17]. The downstream target of SnRK1, the transcription factor bZIP63 transcriptionally regulated the genes encoding for starch degrading enzymes STARCH EXCESS1 (SEX1), phosphoglucan, water dikinase (PWD) and disproportionating enzyme 2 (DPE2), leading to faster starch degradation during the night [18]. Interestingly, T6P, SnRK1 and bZIP63 convey the sugar status into the circadian oscillator [19]. Indeed, bZIP63 directly regulates the transcription of the *PSEUDO RESPONSE REGULATOR7* (*PRR*7) gene, which is involved in the circadian oscillator and is highly expressed in the morning [19]. These players illustrate the complexity and interplay between sugar sensing, starch metabolism and circadian clock.

Experimentally, mutant analyses have been used to investigate the functional roles of specific genes involved in sugar sensing, starch metabolism and the circadian clock. However, to understand the dynamic nature of starch metabolism adjustment, it is necessary to develop a framework capable of assessing the impacts of genetic defects in specific pathways on the overall system-level behaviour of starch metabolism. To achieve this, the use of a mathematical model that formalizes the interplay between sugar sensing, starch metabolism and circadian clock becomes advantageous. Several mathematical models have been proposed to explain the potential mechanism for rapid adjustment of the rate of starch degradation in response to photoperiod [20]. Among these models, the carbon homeostasis model [21,22] stands out as ideal for assessing the impacts of genetic defects in sugar sensing, starch metabolism, and the circadian clock. This model captures the dynamic regulation of starch metabolism by the circadian clock and sugar status in a simple yet comprehensive manner, allowing for model fitting to starch metabolism data derived from various loss-of-function mutants.

This study aims to evaluate the impact of genetic defects on potential regulatory processes of starch metabolism at the system level. To this aim, we first obtained starch metabolism data using both wild-type *Arabidopsis* plants and 10 different mutants affecting clock components, sugar sensing pathways and starch-degrading enzymes. By leveraging the experimental data obtained in this study, we fitted the carbon homeostasis model to the data. This allowed us to assess how each parameter value of the model was affected by genetic defects in each regulatory pathway. Through these system-level assessments of the genetic defects on starch metabolism, we demonstrate the significant role of synergistic regulation from the circadian clock and photosynthetic sugars in optimizing the starch degradation process within a fluctuating photoperiodic environment. This framework facilitates the study of starch metabolism dynamics and its regulation by the circadian clock and sugar signalling pathways at a system level, contributing to a deeper understanding of the complex interplay within this biological system.

## 2. Methods

### 2.1. Summary of the carbon homeostasis model

**Figure 1.**
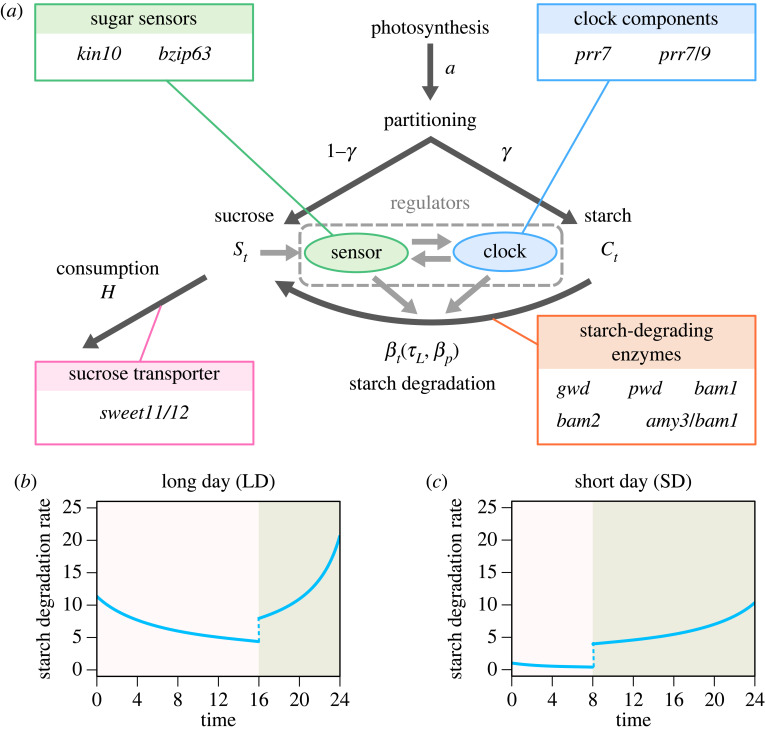
The carbon homeostasis model and the candidate genes involved in each pathway of starch metabolism. (*a*) An illustration of carbon homeostasis model and *Arabidopsis* mutants used in this study. (*b,c*) The diel profile of starch degradation rate predicted by the carbon homeostasis model based on the achievement of carbon homeostasis under given photoperiodic conditions. (*b*) is the predicted profile of the starch degradation rate adjusted to the long-day (LD) condition and (*c*) adjusted to the short-day (SD) condition. It reaches the peak level at dawn, and the timing of the trough is determined by the subjective photoperiod *τ_L_*. When the starch degradation is perfectly adjusted to the given photoperiod, *τ_L_* coincides with the actual photoperiod.

To assess the impacts of genetic defects on starch metabolism, we performed an integrated analysis of the time series data of starch metabolisms in *Arabidopsis* plants and the carbon homeostasis model describing the dynamics of starch and sugar in a plant leaf [21–23]. In this subsection, we briefly explain about the model, because understanding the biological meaning of each of model parameters is crucial in this study. The carbon homeostasis model includes two variables: concentrations of starch *C_t_* and sucrose *S_t_*. Carbon is fixed by photosynthesis at a rate *a* and partitioned into starch and sucrose during the light period. The partitioning rate to starch is represented as *γ*, and the increment of starch and sucrose per unit time under light is given as *aγ* and *a*(1–*γ*) respectively (figure 1*a*). The accumulated starch is then broken down at the degradation rate *β_t_*, which is dependent on time of day due to the clock regulation and light–dark cycle of the environment (figure 1*a*).

Because starch forms a granule packed in high density within chloroplasts, starch degradation occurs at the surface of the granule. Thus, starch degradation rate is assumed to be proportional to the surface area of the starch granule, which is represented as *C_t_^κ^*. The value of *κ* depends on the shape of a granule, and we assumed spherical shape, setting *κ* = 2/3. Using this formulation, the amount of starch broken into sucrose per unit time is expressed as $\beta_{t}C_{t}^{\kappa }$. Finally, sucrose is transported to sinks or consumed by respiration at a rate *H* (figure 1*a*). Overall, the dynamics of starch and sucrose concentration is described as the following set of differential equations:

2.1 $$\frac{dC_{t}}{dt}=\{\;\;\begin{array}{c} a\gamma -\beta_{t}C_{t}^{\kappa }\;\;\;\;(\mathrm{under}\;\mathrm{light})\\ -\beta_{t}C_{t}^{\kappa }\;\;\;\;\;\;\;\;\;(\mathrm{under}\;\mathrm{dark}) \end{array}$$

and

2.2 $$\frac{dS_{t}}{dt}=\{\begin{array}{c} \;\;\;a(1-\gamma )+\beta_{t}C_{t}^{\kappa }-HS_{t}\;\;\;(\mathrm{under}\;\mathrm{light})\\ \;\;\;\beta_{t}C_{t}^{\kappa }-HS_{t}\;\;\;\;\;\;\;\;\;\;\;\;\;\;\;\;\;(\mathrm{under}\;\mathrm{dark}) \end{array}.$$

In equation (2.1), time scale is standardized with the length of a day as 1. Previous studies have demonstrated that when temporal variations in sucrose is minimized (i.e. sucrose homeostasis is realized regardless of day–night fluctuation in light condition), the diel profile of starch always becomes linear and vice versa.

2.3 $$\{\;\;\begin{array}{c} \frac{dS}{dt}=0\\ S_{t}=S_{0} \end{array}\Leftrightarrow \frac{dC}{dt}=\{\begin{array}{c} a-HS_{0}\;(\mathrm{under}\;\mathrm{light})\\ -HS_{0}\;\;\;\;\;(\mathrm{under}\;\mathrm{dark}) \end{array}=\mathrm{constant}.$$

As reported in the previous study [22], the shape of starch degradation rate *β_t_* that realizes sucrose homeostasis is predicted to be a nonlinear function of time that has a peak at dawn (figure 1*b,c*), as formalized as

2.4 $$\beta_{t}(\beta_{p},\tau_{L})=\{\;\;\begin{array}{c} \frac{a(\gamma -1+\tau_{L})\beta_{p}^{\kappa }}{{\left\{a\beta_{p}(1-\tau_{L})t+1\right\}}^{\kappa }}\;\;(\mathrm{under}\;\mathrm{light})\\ \frac{a\tau_{L}\beta_{p}^{\kappa }}{{\left\{a\beta_{p}\tau_{L}(1-t)+1\right\}}^{\kappa }}\;\;(\mathrm{under}\;\mathrm{dark}) \end{array}.$$

The starch degradation function (equation (2.4)) has two parameters *β_p_* and *τ_L_*. *β_p_* represents the degradation potential, which determines the maximum starch degradation rate at dawn. When *β_p_* is small, starch degradation rate is low (electronic supplementary material, figure S1), and starch is likely to remain after dawn. Subjective photoperiod *τ_L_* represents the diel cycle of the starch degradation rate. In this model, it is assumed that plants adjust their starch degradation rate so that sucrose concentration becomes constant through a day–night cycle, which leads to the synchronization of the starch degradation profile with the external day–night cycles [22]. When the sucrose concentration is perfectly constant, the subjective photoperiod *τ_L_* becomes equal to the external one and when the sucrose is depleted or excessed due to the excessive or reduced starch degradation, the *τ_L_* becomes longer or shorter than the external photoperiod, respectively. Therefore, *τ_L_* reflects how accurately the diel profile of starch degradation is synchronized with the external photoperiod. Here, we examine whether such an adjustment is observed in real plants by estimating and comparing the values of *τ_L_* in *Arabidopsis* plants under short and long photoperiod. Note that Seki *et al*. [22] demonstrated that if the light period is too short, the sucrose homeostasis cannot be maintained perfectly due to a severe limitation of light energy, photosynthesis and consequently carbon partitioning, and they predicted that, in such short photoperiod, starch degradation rate (*β_t_*) becomes 0 under light period to minimize the discrepancy from the carbon homeostasis [22]. Therefore, we assumed *β_t_* becomes 0 under light period in extremely short photoperiod. The impact of changes in each parameter value on *β_t_*, *C_t_* and *S_t_* is illustrated in electronic supplementary material, figure S1.

### 2.2. Data acquisition of starch metabolism in *Arabidopsis* plants

Here we explain the methods to obtain starch metabolism data used to assess the impacts of genetic defects on starch metabolism. We measured maltose, sucrose, and starch from wild-type and 10 mutants of *A. thaliana* (figure 1*b*) under long- and short-day conditions. The plant lines used in this study are listed in table 1. *Arabidopsis* seeds were spread onto 12 cm pots filled with *Arabidopsis* basic medium composed mainly of white peat, pre-soaked with 0.15% Previcur (fungicide) and 0.10% boron in water and placed at 4°C in the dark for 3 days to synchronize seed germination. After stratification, the pots were transferred into a controlled growth chamber, in short day (SD; 8 h light / 16 h dark, 180 µmol m^−2^ s^−1^ irradiance) or long day (LD; 16 h light/8 h dark, 120 µmol m^−2^ s^−1^ irradiance) at a temperature of 21/19°C light/dark cycles and relative humidity of 75%. The differences in irradiance used in SD and LD conditions aim to minimize differences in plant developmental stages at the time of the harvest. The whole rosette was harvested 30 days after sowing at ZT0, 1, 2, 4, 8, 12, 15, 16, 20, 23 and 24 for LD and ZT0, 1, 2, 4, 7, 8, 12, 16, 20, 23 and 24 for SD, snapped freeze into liquid nitrogen and stored at −80°C until processing. Pools of three and eleven rosettes were harvested to constitute a biological replicate for LD and SD, respectively. In total, three or five biological replicates were collected for plants growing in SD and LD conditions, respectively. Fifty milligrams of frozen ground rosettes were used for starch and metabolite quantification as described by da Silva *et al*. [30]. Maltose and sucrose were quantified using standard curve.

**Table 1.** List of the mutants used for the analysis.

mutant name	gene name	AGI code	function	group	references
*gwd*	*GWD*	AT1G10760	starch phosphorylation	I	[24]
*pwd*	*PWD*	AT4G24450	starch phosphorylation	I	[25]
*bam1*	*BAM1*	AT3G23920	starch hydrolysis	I	[26]
*bam2*	*BAM2*	AT4G00490	starch hydrolysis	I	[27]
*amy3/bam1*	*AMY3/BAM1*	At3g23920/ At1g69830	starch hydrolysis	I	[28]
*sweet11/12*	*SWEET11/12*	AT3G48740/AT5G23660	sucrose transporter	II	[12]
*prr7*	*PRR7*	At5g02810	sugar-sensitive clock component	III	[29]
*prr7/9*	*PRR7/9*	At5g02810/ AT2G46790	clock component the double mutant with extremely longer circadian period	III	[10]
*kin10*	*KIN10*	AT3G01090	central regulator of C homeostasis (catalytic subunit of SnRK1)	IV	[16]
*bzip63-1*	*bZIP63*	At5g28770	sugar-sensitive transcription factor downstream of SnRK1	IV	[18]

### 2.3. Mutant information

In order to evaluate the effectiveness of our methodology using the carbon homeostasis model, we specifically chose 10 mutants that are potentially associated with each model parameter based on previous studies (figure 1*a*). Our mutants are involved in starch turnover, sucrose transportation, the circadian clock and the sugar sensing pathway (figure 1*a*). We grouped the 10 mutants into four categories and explain the function of each gene within each group.

#### 2.3.1. Group I: starch-degrading enzymes

The group includes five mutants whose function is associated with the starch degradation: *gwd*, *pwd*, *bam1*, *bam2* and *amy3/bam1*. *gwd* and *pwd* are the mutants of the starch phosphorylation enzymes, glucan, water dikinase (*GWD*)[24] and *PWD* [25]. These enzymes catalyse the phosphorylation of the glucan chain on the surface of starch granules, the first step of the starch degradation [2,31,32]. The importance of the phosphorylation step in regulation of starch degradation has been proposed by experimental and theoretical studies [20]. *bam1* and *bam2* are the *β*-amylases that catalyse the hydrolysis of the oligosaccharides, which produces maltose [26,27]. The expression of BAM1 and BAM2 has been known as circadian-regulated. *amy3/bam1* is a double mutant of the α-amylase and β-amylase. AMY3 and BAM1 are mainly localized in guard cells and regulating stomatal opening in response to light environment [28].

#### 2.3.2. Group II: sucrose transporter

The group includes the mutant associated with the sucrose flux in a leaf. *sweet11/12* is the mutant of the sucrose transporters *SWEET11* and *SWEET12* which plays a significant role in the phloem loading of source–sink transportation in plants [12,33]. *sweet11/12* shows significantly higher sucrose concentration in a leaf tissue than wild-type and it does not oscillate between day and night. Because in the carbon homeostasis model, plants are assumed to sense the deviation of sugar concentration rather than the concentration itself to synchronize the starch degradation to the external diel cycle, the phenotype of this mutant, high concentration but relatively small deviation of sugar, is suitable for our analysis, compared with other sucrose transporters such as sucrose-H^+^ cotransporters (SUCs) [33]. These genes are also known to be regulated by the circadian clock [34].

#### 2.3.3. Group III: clock components

The group includes the mutants of the sugar-sensitive components of the circadian clock: *prr7* and *prr7/9*. The transcription of *PRR7* is known to be sensitive to sugar concentration and the main candidate of the sugar-dependent phase modulation of the circadian clock [19,29]. The double mutant *prr7/9* has extremely longer circadian photoperiod [10]. It has been demonstrated that *prr7*/*9* delays the onset of starch degradation [9]. These genes may correspond to the model parameters (*τ_L_* and *β_p_*) which reflect the time-dependent profile of starch degradation (figure 1*b*; electronic supplementary material, figure S1). Particularly, we can test the contribution of the clock regulation for the adjustment of starch degradation *β_t_*.

#### 2.3.4. Group IV: sugar sensing

This group includes the mutant associated with the sugar sensing pathways particularly involved in interaction between sugars and clock: *kin10* and *bzip63-1*. *KIN10* is a catalytic subunit of Snf1-related protein kinase (SnRK1) and *bZIP63* is a sugar-sensitive transcription factor that regulates many pathways including the transcript level of the starch degrading enzymes. These genes are involved in the sugar-dependent phase modulation of the circadian clock, in which SnRK1 activates bZIP63 under starvation and bZIP63 transcriptionally regulates the expression of PRR7 [18,19]. If the sugar sensing is the major contributor for the adjustment of starch degradation to external photoperiods, these mutants may show the defects in the subjective photoperiod *τ_L_* (figure 1*b,c*)

### 2.4. Estimation of starch degradation rate *β_t_* using the maltose time series data

The previous theoretical study predicted that the starch degradation rate *β_t_* exhibits diel fluctuations with a peak at dawn [22]. By using time series data for maltose and starch content, we can estimate the starch degradation rate and compare it with the prediction from the model. Maltose is a product of starch breakdown. Because the reaction of starch degradation occurs on the surface of the starch granules, the concentration of maltose is proportionate to the reaction area of starch degradation, which can be approximated by the volume of starch raised to the power of 2/3. Thus, the starch degradation rate *β_t_* can be calculated by dividing maltose concentration by the surface area of starch granule as follows:

2.5 $$\beta_{t}≅\frac{\left[\mathrm{Maltose}\right]}{{\left[\mathrm{Starch}\right]}^{2/3}}\;.$$

We compared observed and predicted starch degradation rates by examining a decreasing trend during the light period and an increasing trend during the dark period in a diurnal cycle. As the estimate of *β_t_* is a relative value, the scale of *β_t_* measured in maltose data and that measured in the model setting may not always align. To account for this discrepancy, we standardized both empirical and theoretical estimates so that their maximum values equal 1. The measured concentration of maltose is displayed in electronic supplementary material, figure S2.

### 2.5. Model fitting to the data of starch and sucrose concentration in *Arabidopsis* plants

To fit the model to time series data of starch and sucrose, we applied a Bayesian estimation. The time series data ***X*** = {*X*_1_, *X*_2_, … , *X_n_*} includes four variables at each time point *t* that range from 1 to *n*,

2.6 $$Xt=(C_{t}^{LD},S_{t}^{LD},C_{t}^{SD},S_{t}^{SD}),$$

where $C_{t}^{L}$ and $S_{t}^{L}$ stand for starch and sucrose level at time *t* and superscript *L* = LD or SD indicates long- or short-day condition, respectively. Our primary objective is to find the probability density of the model parameters

2.7 $$\theta =(a,\gamma ,H^{LD},\tau_{L}^{LD},H^{SD},\tau_{L}^{SD},\beta_{p}),$$

by fitting the model to the observed data. We consider that sucrose consumption rate (*H*) depends on photoperiod because previous studies demonstrate that photoperiod affects the export of C from the source leaves [35]. We also estimated *τ_L_* in LD and SD respectively to examine whether its value is adjusted by photoperiod in *Arabidopsis* plants.

The Bayesian framework practically implements the posterior inference of parameters by incorporating prior knowledge through a tailored likelihood function. The Bayesian inference is generally grounded on Bayes' theorem as follows:

2.8 $$P(\theta |X,M)=\frac{P(\theta )P(X|\theta ,M)}{P(X)}\propto P(\theta )P(X|\theta ,M),$$

where *P*(***θ***|***X***, *M*) represents the posterior probability distribution of the parameter vector ***θ***, given the model *M* and observed data ***X***. *P*(***X|θ***, *M*) represents the likelihood function, and *P*(***X***) represents the probability distribution of observed data. *P*(***θ***) is the prior probability distribution of the parameters, determined by prior knowledge about the parameter before the experiment is conducted. Bayesian inference based on the Markov chain Monte Carlo (MCMC) algorithm approximates the sampling of the parameter from the target posterior probability distribution *P*(***θ***|***X***, *M*) by utilizing the information of *P*(***θ***)*P*(***X|θ***, *M*). From the samples generated by the MCMC algorithm, we can estimate the posterior probability distribution of the parameters and compute posterior quantities such as the posterior mean, variance and other relevant statistics.

The likelihood *P*(***X|θ***, *M*), which manifests the probability of observing the actual data, is computed by assuming that measurement noise follows a normal distribution with mean ***μ****_t_* and variance *σ*^2^. Here, ***μ****_t_* is the true concentration of starch and sucrose in a leaf tissue under a specific time and photoperiod, which is derived from the model *M* with a given set of parameters ***θ***. It is assumed that the variance *σ*^2^ differs between starch and sucrose and is denoted as $\sigma_{c}^{2}$ and $\sigma_{s}^{2}$ for starch and sucrose, respectively. The true concentration ***μ****_t_* is calculated by solving the differential equations defined above numerically using the fourth order Runge–Kutta method. The initial condition was set as C(0) = 1/*β_p_* and S(0) = *aτ_L_*/*H*, which is the steady solution when the starch degradation is perfectly adjusted [22]. We calculated the ***μ****_t_* for a duration equivalent to six days and used the latter three-day portion of it for the model fitting to mitigate the impact of the initial condition.

As we lack prior information about the specific parameter values, we employed a uniform distribution for *P*(***θ***). From the biological constraints, we restricted the range of the parameters to be positive, specifically between 0 and 1 for the parameters associated with the ratios. Given *P*(***θ***)*P*(***X|θ***, *M*), we applied the Hamiltonian Monte Carlo (HMC) method through the use of a probabilistic programming language, Stan (Rstan v. 2.21.3) to obtain samples of parameters drawn from the posterior distribution. We ran four independent Markov chains, initialized at distinct random starting points, with a burn-in phase lasting 1000 iterations, followed by a sampling phase of an additional 1000 iterations. After computing the posterior distribution of each parameter, we assessed the convergence and robustness of the distribution by evaluating the maximum R hat and effective sample size, as recommended by the Stan reference guide. We also computed the point estimate of the parameter value from the posterior samples. We adapted the maximum *a posteriori* (MAP) estimate as the point estimate. To do this, we first applied kernel density estimation to the posterior samples using the ‘density’ function of R software (v. 4.1.2 (2021-11-01)) and then calculated the MAP estimates as the values corresponding to the maximum density. Our model fitting to the time series data of wild-type was performed independently for 13 batches.

### 2.6. Model selection

To estimate the parameter values of the model for each mutant, we assume that values of all parameters are not necessarily different between the wild-type and the mutant. Rather, some parameter values are equivalent between the wild-type and the mutant because of conserved genetic background. In this context, we assume that the vector representing the parameter values for the mutant (***θ****_mt_***)** is given as the sum of two terms: the estimate for the wild-type (***θ****_wt_*) and the deviation from the wild-type (**Δ**). Here, we used the posterior estimates for ***θ****_wt_*. If the parameter value for the mutant does not deviate significantly from that of the wild-type, the respective element of the vector **Δ** equals to 0. Given that our model has seven parameters, the number of possible combinations of elements of **Δ** with zero values is 2^7^ = 128. We treated these combinations as distinct models and select the optimal model by evaluating the widely applicable information criterion (WAIC). After estimating the posterior probability distribution of a mutant through Bayesian estimation for each model, we calculated WAIC using the following formula:

2.9 $$\mathrm{WAIC}=\;-2\overset{N}{\underset{i=1}{\sum }}\log\left(\frac{1}{M}\overset{M}{\underset{\;j=1}{\sum }}P(X_{i}|\theta_{j})\;\right)+2\overset{N}{\underset{i=1}{\sum }}(\mathrm{Var}(\log P(X_{i}|\theta_{j})))\;.$$

*θ_j_* is a sampled parameter from the estimated posterior distribution (HMC sample). The first term is a sum of mean likelihood for data *X_i_* with parameter sample *θ_j_* from posterior distribution. The second term is the sum of the variance of the likelihood. The model with the lowest WAIC was selected as the optimal model.

### 2.7. Assessing the impact of genetic defects on starch metabolism

To evaluate the impact of genetic defects on starch metabolism we compared the estimated parameter values between the mutant and the wild-type. We calculated the MAP estimates from MCMC samples for each parameter and for each mutant. Then, from the posterior estimate, we computed log2 foldchange from wild-type for each mutant

2.10 $$\Delta \theta \;:=\log_{2}\left(\frac{{\left(\theta \right)}_{\mathrm{mutant}}^{*}}{{\left(\theta \right)}_{wt}^{*}}\right),$$

and extracted the mutants with |Δ*θ*|≧1.5 as those displaying significant effects. Additionally, to evaluate the effect of genetic defects on subjective photoperiod ($\tau_{L}^{LD}$ and $\tau_{L}^{SD}$), we define Δ*τ_L_* as follows:

2.11 $$\Delta \tau_{L}^{LD}\;:={\left(\tau_{L}^{LD}\right)}_{\mathrm{mutant}}-{\left(\tau_{L}^{LD}\right)}_{wt}$$

and

2.12 $$\Delta \tau_{L}^{SD}\;:={\left(\tau_{L}^{SD}\right)}_{\mathrm{mutant}}-{\left(\tau_{L}^{SD}\right)}_{wt},$$

where (*τ_L_*)_mutant_ and (*τ_L_*)*_wt_* are the estimated values of the subjective photoperiods in the mutants and wild-type, respectively. We numerically calculated the distribution of Δ*τ_L_* by extracting *τ_L_* randomly from the MCMC samples of the wild-type and the mutants. The negative sign of the $\Delta \tau_{L}^{LD}$ indicates that the mutant has a decreased ability to lengthen the subjective photoperiod compared with the wild-type in the long-day condition. Conversely, the positive sign of the $\Delta \tau_{L}^{SD}$ signifies that the mutant has a decreased ability to shorten the subjective photoperiod compared with the wild-type in the short-day condition.

## 3. Results

### 3.1. Fitting the carbon homeostasis model to the wild-type data

**Figure 2.**
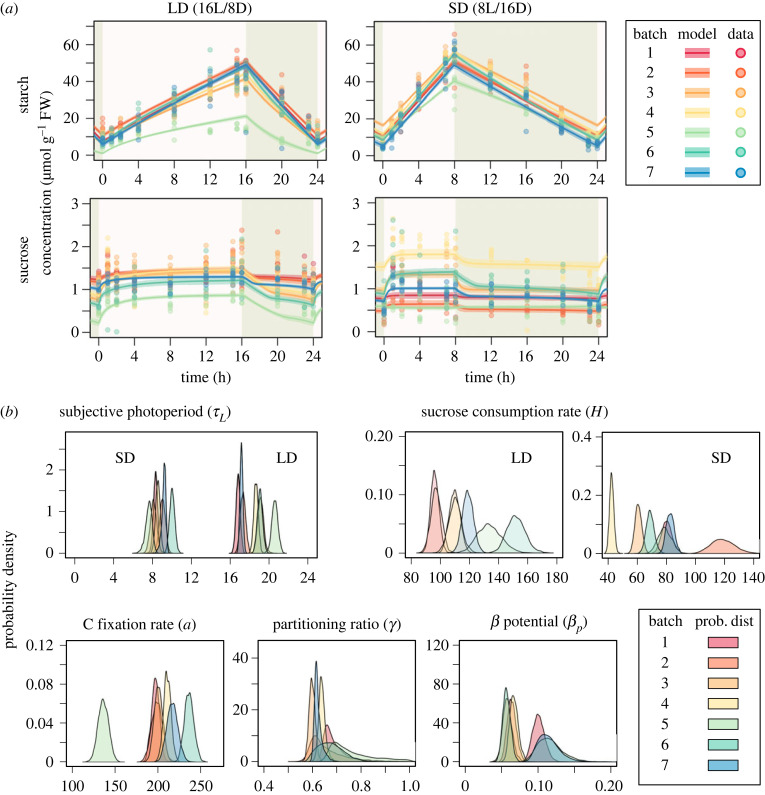
The result of model fitting using starch and sucrose data in wild-type. (*a*) Comparison between model prediction and observation. Solid line and transparent band indicate the mean and 95% high density interval (HDI) of the model prediction at each time point respectively. The dots represent the observed data, with each colour denoting a distinct batch. FW: formula weight. (*b*) Density plots illustrating the estimated distribution of parameters. Each colour and accompanying number correspond to a specific batch. The estimated distributions of parameters exhibited slight variations among batches, reflecting the differential dynamics of starch and sucrose in *Arabidopsis* plants across different batches (i.e. the slope of starch increase and decrease and the level of sucrose concentration in figure 2*a*).

To evaluate the impact of genetic defects on potential regulatory processes of starch metabolism at the system level, we first applied the carbon homeostasis model to the wild-type data. Model fitting to the starch and sucrose data of wild-type was overall successful. The prediction of the model depicted more rapid accumulation and slower degradation of starch under SD compared with those under LD, which was consistent with the observations (figure 2*a*). The measured levels of starch and sucrose differed from batch to batch, possibly due to subtle differences in experimental condition or material preparation that could not be controlled. The carbon homeostasis model successfully explained these variations, which were mainly reflected as variations in photosynthetic activity (*a*) and sucrose consumption parameter values (*H^LD^* and *H^SD^*).

The ability of the adjustment of starch degradation in response to photoperiod changes in wild-type plants was evaluated by comparing the estimated values of the subjective photoperiod parameter *τ_L_* between LD and SD condition. If starch degradation rate is properly adjusted to realize the C homeostasis, the value of *τ*_L_ should equal to the external photoperiod. We found that the estimated subjective photoperiods under LD ($\tau_{L}^{LD}$) and SD conditions ($\tau_{L}^{SD}$) were distributed in the ranges close to the external photoperiods (figure 2*b*), suggesting that wild-type plants properly adjusted their starch degradation rates to maintain the C homeostasis. In SD conditions, mean of estimated values for $\tau_{L}^{SD}$ ranged from 7.2 to 9.6 h across batches, which encompassed the external photoperiod of 8 h. Conversely, under LD conditions, mean of estimated values for $\tau_{L}^{LD}$ ranged from 16.8 to 19.2 h, which was slightly longer than the external photoperiod of 16 h. Additionally, the sucrose consumption rate (*H*) exhibited notable differences between LD and SD conditions (figure 2*b*), with higher values observed under LD compared with SD conditions.

**Figure 3.**
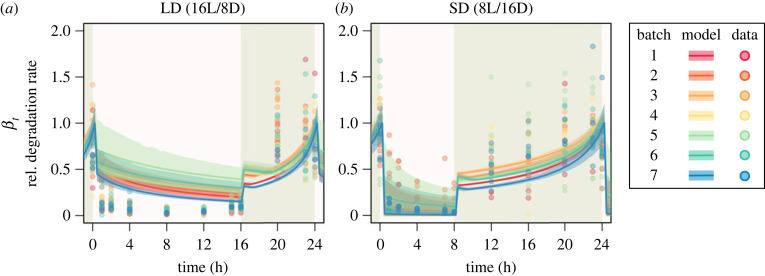
Comparison of predicted and observed starch degradation rate *β_t_*. Each colour corresponds to a specific batch. The solid line and transparent band indicate the mean and 95% HDI of the model prediction at each time point, respectively. The dots indicate the empirically estimated *β_t_* based on measurement of maltose and starch contents.

Using the estimated parameters, we can predict the diel profiles of the starch degradation rates *β_t_* of wild-type plants given in equation (2.4). The predicted diel profile of starch degradation rate has a sharp peak at dawn (figure 3). To validate this prediction, we estimated starch degradation rate experimentally by measuring maltose concentration (see Methods). Experimentally estimated profile of starch degradation rate, $\beta_{t}^{\exp}$, demonstrated similar trends to the predicted one, such as decline during the light period and an increase during the dark periods, under both LD and SD conditions (figure 3), which is in agreement with the prediction from the carbon homeostasis model. Performance of model fitting was slightly better under SD conditions (average mean absolute error (MAE) among batches = 0.4305 ± 0.0346) compared with the LD condition (0.5367 ± 0.0664) (electronic supplementary material, table S2).

### 3.2. Impacts of genetic defects on starch degradation and sucrose consumption

**Figure 4.**
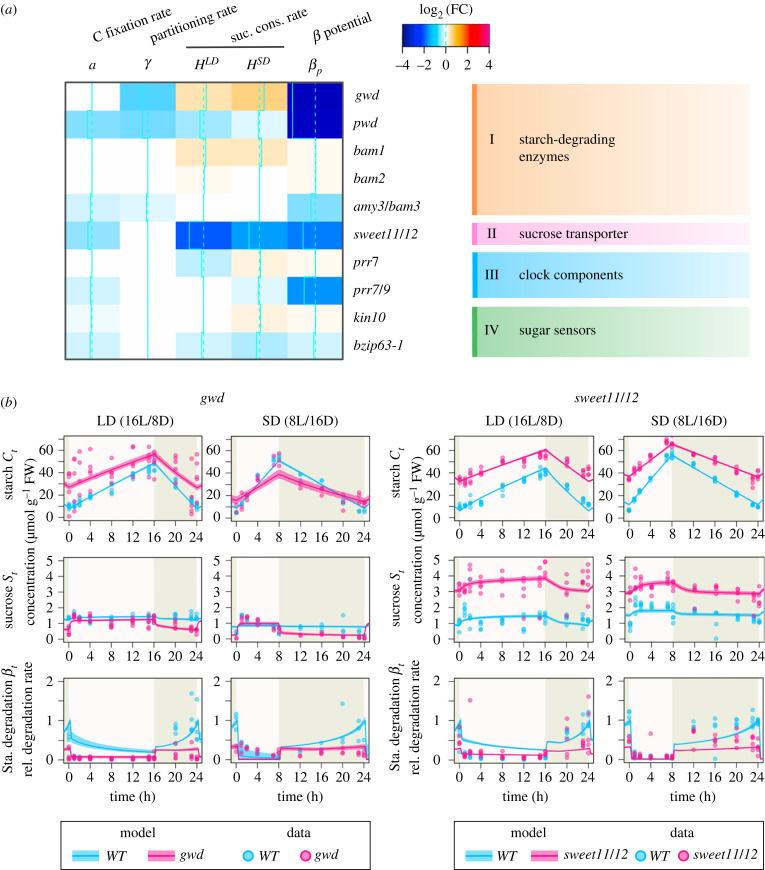
Changes in carbon flux and starch degradation parameters in mutants. (*a*) Heatmap showing log2 fold changes in parameters *a*, *γ*, *H* and *β_p_*. White cells indicate parameters with identical values between the wild-type and the respective mutant. (*b*) Model prediction and experimental data of starch and sucrose contents in *gwd* and *sweet11/12,* characterized by impairments in starch degradation and sucrose flux. The solid line and transparent band represent the mean and 95% HDI of the model predictions at each time point respectively. The dots indicate the experimental data.

To assess the impacts of the genetic defects on starch metabolism using the carbon homeostasis model, we next performed the model fitting to the data obtained from the mutants potentially associated with each model parameter according to previous studies. The mutants were included in the following four groups: (I) starch-degrading enzymes, (II) sucrose transporter, (III) clock components, and (IV) sugar sensing (table 1, figure 1*b*). To avoid over-fitting and to characterize the phenotypes with a small set of parameters, we first applied model selection (see Methods). The analysis of model selection revealed that most of the physiological processes associated with starch metabolism are affected by defects in at least three of the 10 genes examined here (figure 4*a*; electronic supplementary material, table S1).

Across seven parameters of the carbon homeostasis model, the most sensitive parameter to the genetic defect was the starch degrading potential *β_p_* which controls the maximum level of the starch degradation rate. It was affected by the defect of most of genes from all groups (figure 4*a*). The starch phosphorylation enzymes *gwd* and *pwd* exhibited a marked deviation from the wild-type in *β_p_*, and the starch degradation rate *β_t_* was estimated to be low throughout the day without a peak around dawn observed in the wild-type (figure 4*b*). By contrast, other degradation enzymes, *bam1*, *bam2* and *amy3*/*bam1*, have little effects on *β_p_* compared with *gwd* and *pwd* (figure 4*a*). It indicates that the impact on starch degradation rate varies even among the starch degrading enzymes. Additionally, in a mutant of sugar-sensitive transcription factor *bzip63-1*, the starch degradation rate and starch degradation potential (*β_p_*) slightly decreased compared with the wild-type (figure 4*a*). By contrast, *prr7,* one of the regulatory targets of *bZIP63*, did not show such a decrease in starch degradation (figure 4*a*).

**Figure 5.**
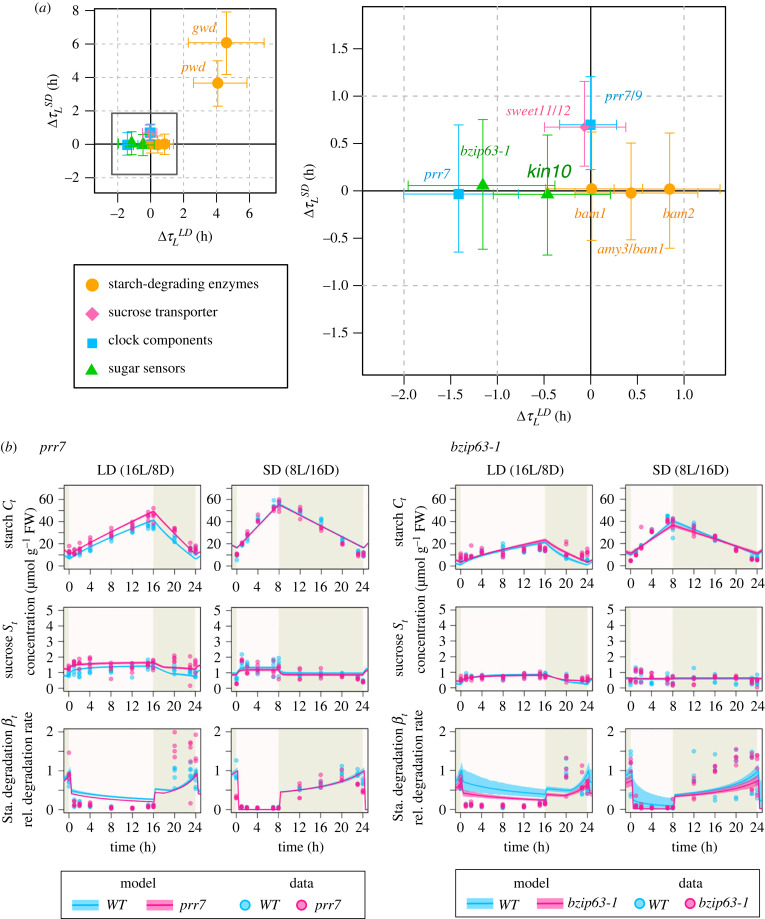
Defects in adjustment of the starch metabolism in response to changing photoperiods, along with starch and sucrose profile of the circadian clock and sugar sensing mutants. (*a*) Deviation of the mutants' subjective photoperiod from the wild-type (*Δτ_L_*). The x-axis represents *Δτ_L_* under $LD(\Delta \tau_{L}^{LD})$, while the y-axis represents *Δτ_L_* under SD condition ($\Delta \tau_{L}^{SD}$). The colour-coded dots indicate the functional group of each gene. Negative values along the $\Delta \tau_{L}^{LD}$ axis and positive values along the $\Delta \tau_{L}^{SD}$ axis indicate reduced responsiveness to LD and SD conditions, respectively. (*b*) Model prediction and experimental data illustrating starch and sucrose dynamics in *prr7* and *bzip63-1* mutants, which are involved in the sugar-dependent phase modulation of the circadian clock. The solid line and transparent band represent the mean and 95% high density interval of the model predictions at each time point, respectively, while the dots represent the experimental data.

The sucrose transporter *sweet11/12* showed excessive starch accumulation and high concentration of sucrose (figure 4*b*). The estimated sucrose consumption rate *H* was extremely lower than the one in the wild-type in both photoperiods (figure 4*a*), which is in agreement with its molecular function as a sugar transporter that exports sugars from source to sink organs. In addition to the sucrose consumption, starch degradation rate was also estimated to be lower in *sweet11/12* (figure 4*b*), which is consistent with the previous experiments, showing that the starch degradation is inhibited by sucrose via T6P signalling [13] and by glucose produced from the hydrolysis of sucrose in a hexokinase (HXK)-dependent manner[36]. Carbon partitioning rate (*γ*) was less sensitive to the genetic defect because only three mutants in the Group I (starch-degrading enzymes) showed diminished values compared with the wild-type (figure 5*a*; electronic supplementary material, table S1). The photosynthesis rate (*a*) was also less sensitive to the genetic defects (|log_2_(*θ*_mutant_ /*θ*_wt_) | < 1.5) (figure 4*a*).

### 3.3. Genetic defects in circadian clock components and sugar sensing pathway revealed significant impacts on the regulation of starch degradation under long-day photoperiod

To assess the ability of the adjustment of starch degradation rate from the system-level perspective, we focused on the subjective photoperiod as we did above for wild-type plants. Because the subjective photoperiod reflects the state of synchronization of starch degradation profile to the external photoperiods, we calculated the difference of the subjective photoperiods between mutants and wild-type plants, $\Delta \tau_{L}^{LD}$ and $\Delta \tau_{L}^{SD}$ (see Methods). Larger deviation of the subjective photoperiods indicates that the mutant has a defect in controlling the synchronization process from the view of the C homeostasis. The top two mutants that showed the largest deviation from the wild-type were *gwd* and *pwd* (4.3 and 4.0 h in LD and, 5.9 and 3.6 h in SD, respectively; figure 5*a*), which can be explained by the direct role of these enzymes in starch degradation, rather than the regulatory role of the enzyme activity for starch degradation. In contrast to *gwd* and *pwd*, other starch degradation enzymes had less effects on the response to photoperiod (figure 5*a*). Among the other candidate mutants for the regulatory roles in starch degradation, the top two mutants that showed significant deviation from the wild-type were a sugar-sensitive clock component *prr7* (−1.4 h) and its transcriptional regulator *bzip63-1* (−1.1 h) (figure 5*a*; electronic supplementary material, table S1). These mutants exhibited a subjective photoperiod that was estimated to be more than 1 h shorter ($\Delta \tau_{L}^{LD}$ > 1 h) compared with the wild-type. On the other hand, another mutant involved in the sugar-induced modulation of the circadian clock, *kin10*, had a relatively smaller impact on the subjective photoperiod ($\Delta \tau_{L}^{LD}$ < 1 h).

## 4. Discussion

**Figure 6.**
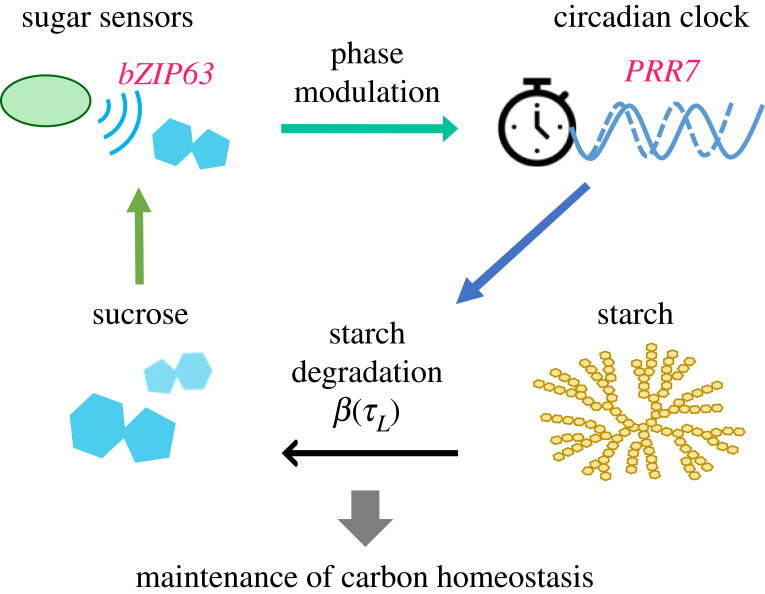
Summary of our findings. Our system-level assessment revealed the major contribution of the synergetic interplay between *bZIP63* involved in the sugar sensing pathway and *PRR7* involved in the circadian clock to the regulation of starch degradation for maintenance of C homeostasis.

We used a carbon homeostasis model that formalizes the interplay between sugar sensing, starch metabolism and the circadian clock to assess the impacts of genetic defects in specific pathways on the overall system-level behaviour of starch metabolism. Our system-level assessment revealed that genetic defects in both the sugar sensing pathway (*bzip63*) and the circadian clock (*prr7*) affect the proper adjustment of the starch degradation rate (figures 5*b* and 6). *bZIP63* is a sugar-sensitive transcription factor that regulates the expression of *PRR7* [18,19], thereby modulating the circadian clock phase in a sugar-dependent manner (figure 6). Our analyses predicted that loss-of-function mutations in *bZIP63* and *PRR7* result in the desynchronization of subjective and external photoperiods, with a shorter subjective photoperiod compared with the external photoperiod under LD conditions. This desynchronization between subjective and external photoperiods is predicted to disrupt carbon homeostasis, resulting in impaired growth under changing photoperiod conditions. This result not only confirmed the previous empirical finding that *PRR7* and *bZIP63* are major players in the circadian clock entrainment by photosynthetic sugar [18,19] but also provides a system-level understanding of the dynamic nature of starch metabolism adjustment. While we have examined a limited number of mutants in this study, if data on sugar metabolism profiles in various types of mutants become available in the future, we will be able to assess the impacts of genetic defects in each component of the full gene regulatory network.

We also found that loss-of-function mutations in *bZIP63* and *PRR7* have a greater impact on the subjective photoperiod under LD conditions compared with SD conditions (figure 5*a*). The desynchronization between the subjective and external photoperiod was predicted only under LD conditions but not for SD conditions. This suggests that the response to SD conditions is more robust and more tightly regulated compared with the one to LD conditions, probably due to the higher requirement for carbon homeostasis under SD conditions, where there is a greater risk of carbon depletion. This robustness to the shorter photoperiods is consistent with the observation that the response of the clock transcript abondance is restricted to the extremely low light condition or low CO_2_ condition [37]. Furthermore, it has been demonstrated that response of the global gene expression pattern to low sugar levels during the night is more sensitive than high levels in the light, which also supports our result [38]. In addition, it has been known that several mutants of starch biosynthesis or degradation show the growth phenotype rescue under continuous light condition [39], which indicates regulation of starch metabolism is not always necessary for plants' growth when sufficient energy is available because of long photoperiods. To explore the general relationship between the photoperiod-dependent requirement of carbon homeostasis and the subjective photoperiod, future experiments that encompass a wider range of photoperiod conditions are necessary.

Our findings highlight the specialized role of the clock-phase modulation in the regulation of starch degradation in response to changes of the photoperiods. The mutant of *PRR7* only showed the defects in the subjective photoperiod *τ_L_*, which corresponds to the timing of trough in diel oscillation of starch degradation, whereas it did not show significant changes in the other parameters including *β_p_*, which represents the peak level of starch degradation. This result indicates that the phase modulation of the clock is specific to temporal control of the starch degradation for adjusting the timing of trough and peak of the oscillation pattern to the photoperiod of the surrounding environment. On the other hand, *bZIP63*, an upstream regulator of *PRR7*, showed not only the defects in the subjective photoperiod but also the slight decrease of the degradation potential *β_p_*. This may suggest that *bZIP63* also regulates the activity of starch-degrading enzymes in different ways as well as the transcriptional regulation of *PRR7*. This suggestion is in line with an observation that the *bzip63* mutant shows reduced activation of starch-degrading enzyme GWD [18].

Our approach integrating the mathematical model and mutant experiments enabled us to quantify the impacts of genetic defects on the adjustment of starch degradation. The approach can be extended to analyse the data collected in different genetic background and different environmental conditions such as natural environments where photoperiod and light intensity change continuously. Future research may uncover limitations and areas for improvement in this approach. Application of this study to field data would shed new light on the adaptive significance of the feedback regulation of starch metabolism in plants growing in natural conditions.

## Data Availability

The source code and raw data used in this article are available from the GitHub repository: https://github.com/shuichi-kudo/Bayesian_Inference_of_carbon_homeostasi_model [40]. Supplementary material is available online [41].
